# Structure-Morphology-Antimicrobial and Antiviral Activity Relationship in Silver-Containing Nanocomposites Based on Polylactide

**DOI:** 10.3390/molecules27123769

**Published:** 2022-06-11

**Authors:** Valeriy Demchenko, Yevgen Mamunya, Serhii Kobylinskyi, Sergii Riabov, Krystyna Naumenko, Svitlana Zahorodnia, Olga Povnitsa, Nataliya Rybalchenko, Maksym Iurzhenko, Grazyna Adamus, Marek Kowalczuk

**Affiliations:** 1 Department of Polymer Modification, Institute of Macromolecular Chemistry of the National Academy of Sciences of Ukraine, 48. Kharkivske Shose, 02160 Kyiv, Ukraine; ymamunya@ukr.net (Y.M.); kobylinskiy@ukr.net (S.K.); sergii.riabov@gmail.com (S.R.); 4ewip@gmail.com (M.I.); 2 Department of Plastics Welding, Evgeny Oskarovich Paton Electric Welding Institute of the National Academy of Sciences of Ukraine, 11. Kazymyr Malevych Str., 03680 Kyiv, Ukraine; 3 International Polish-Ukrainian Research Laboratory Formation and Characterization of Advanced Polymers and Polymer Composites (ADPOLCOM), Department of Plastics Welding, Evgeny Oskarovich Paton Electric Welding Institute of the National Academy of Sciences of Ukraine, 11. Kazymyr Malevych Str., 03680 Kyiv, Ukraine; gadamus@cmpw-pan.edu.pl; 4 Danylo Kyrylovych Zabolotny Institute of Microbiology and Virology of the National Academy of Sciences of Ukraine, 154. Academic Zabolotny Str., 03680 Kyiv, Ukraine; krystyn.naumenko@gmail.com (K.N.); svetazagorodnya@ukr.net (S.Z.); povnytsa@nas.gov.ua (O.P.); nrybalchenko@ukr.net (N.R.); 5 Laboratory of Biodegradable Materials, Centre of Polymer and Carbon Materials, Polish Academy of Sciences, 34. M. C. Skłodowska St., 41-800 Zabrze, Poland

**Keywords:** polylactide, green tea extract, silver-containing nanocomposite, structure, morphology, thermophysical properties, antimicrobial and antiviral activity

## Abstract

Green synthesis of silver-containing nanocomposites based on polylactide (PLA) was carried out in two ways. With the use of green tea extract, Ag^+^ ions were reduced to silver nanoparticles with their subsequent introduction into the PLA (mechanical method) and Ag^+^ ions were reduced in the polymer matrix of PLA-AgPalmitate (PLA-AgPalm) (in situ method). Structure, morphology and thermophysical properties of nanocomposites PLA-Ag were studied by FTIR spectroscopy, wide-angle X-ray scattering (WAXS), transmission electron microscopy (TEM), thermogravimetric analysis (TGA), and differential scanning calorimetry (DSC) methods. The antimicrobial, antiviral, and cytotoxic properties were studied as well. It was found that the mechanical method provides the average size of silver nanoparticles in the PLA of about 16 nm, while in the formation of samples by the in situ method their average size was 3.7 nm. The strong influence of smaller silver nanoparticles (3.7 nm) on the properties of nanocomposites was revealed, as with increasing nanosilver concentration the heat resistance and glass transition temperature of the samples decreases, while the influence of larger particles (16 nm) on these parameters was not detected. It was shown that silver-containing nanocomposites formed in situ demonstrate antimicrobial activity against gram-positive bacterium *S. aureus*, gram-negative bacteria *E. coli*, *P. aeruginosa*, and the fungal pathogen of *C. albicans*, and the activity of the samples increases with increasing nanoparticle concentration. Silver-containing nanocomposites formed by the mechanical method have not shown antimicrobial activity. The relative antiviral activity of nanocomposites obtained by two methods against influenza A virus, and adenovirus serotype 2 was also revealed. The obtained nanocomposites were not-cytotoxic, and they did not inhibit the viability of MDCK or Hep-2 cell cultures.

## 1. Introduction

Infectious diseases caused by viruses and microorganisms continue to be one of the biggest health problems in the world, despite the rapid progress in drug development and pharmaceutical technology [1,2]. The risk of complications and the spread of infections in society remains high. One of the ways in which viral and bacterial diseases are spread is through contact, as when the source of the spread of microorganisms is a surface that is repeatedly touched by people, such as door handles and dashboards, mobile phones, computer keyboards, and so on. Particularly dangerous in this regard are resistance to the antimicrobial microorganisms *Staphylococcus aureus*, *Escherichia coli*, *Pseudomonas aeruginosa*, fungal pathogen *Candida albicans*, etc. Therefore, there is an urgent need for new antiviral and antimicrobial materials that can protect such surfaces [3]. The most suitable for such purposes are polymer film materials with high protective properties. Another area of application of protective materials is films for biomedical packaging and food packaging [4].

Recently, polymer nanocomposites containing nanoparticles of metals such as silver, copper and zinc oxide have attracted the most attention due to their pronounced pharmacological effects, including antimicrobial, antiviral, anti-inflammatory, immunomodulatory, and high stability in extreme conditions [5,6,7,8,9]. A promising polymer for the creation of antimicrobial and antiviral silver-containing nanocomposites is polylactide (PLA), which has significant advantages over other polymers, namely it is thermoplastic and is characterized by high mechanical strength, biocompatibility and non-toxicity [10,11]. These benefits allow the use of PLAs for biomedical packaging, food packaging, and 3D printing technology.

Additionally, the monomer for the production of PLA may be derived from renewable resources such as corn, sugar beets, sugar cane, wheat, potatoes, rice, and others [12].

In the work of [13], antimicrobial properties of chitosan-Ag polymer composites according to the silver content were studied; the optimal composition of composites was established and on its basis polymeric granules of PLA-Ag-chitosan were developed. At the same time, the size of the synthesized silver nanoparticles was not investigated. Recently, our investigations [14] have shown that thermochemical reduction of Ag^+^ ions in the presence of a synthetic polymer polyethyleneimine (PEI) as a reducing agent and stabilizer occurred above 100 °C for 5 min. The nanocomposites have demonstrated antimicrobial activity against *S. aureus* and *E. coli* strains, and antiviral activity against herpes simplex virus type 1, influenza A virus, and adenovirus serotype 2.

Among the methods of obtaining polymer silver-containing nanocomposites, there are dispersion methods (grinding of silver particles with the subsequent introduction into the polymer matrix) and methods of reduction of Ag^+^ ions directly in the polymer matrix [15]. According to the literature analysis, the reduction of Ag^+^ ions in the polymer matrix is one of the promising ways for obtaining silver-containing polymer nanocomposites, which allows for controlling the size and morphology of nanoparticles [16]. The most common methods of reduction are chemical [17], thermochemical [18], radiation-chemical [19], and the use of plant extracts for reduction [20].

In chemical synthesis, such reducing agents as hydrazine [17], sodium borohydride [21,22], dimethylformamide [23] and others are used. This is a simple and effective way to create nanocomposites with a controlled structure, but its problem is the toxicity of the compounds used. The radiation-chemical reduction of Ag^+^ ions makes it possible to obtain silver-containing nanocomposites without the use of chemical reducing agents, but requires the use of special equipment to generate radiation [16,19]. An effective, inexpensive, and environmentally friendly method of forming silver-containing nanocomposites is thermochemical reduction of silver ions. Still, this method sometimes requires the heating of the films at fairly high temperatures [6,18]. Recently, the method of obtaining silver-containing nanocomposites by reduction of Ag^+^ ions in the presence of polymeric stabilizers using plant extracts (green synthesis) has become widespread [24,25]. Green synthesis through the plant extract is considered to be promising for commercial production of nanoparticles because of its safety, eco-friendliness, cleanliness, and non-toxic approach. Such works are mainly devoted to the studying of nanoparticles in solutions. Nanoparticles with a size of 6.5 to 70 nm were obtained [26,27,28,29].

A review of the literature has shown that so far there are no publications on the study of polymeric silver-containing film nanocomposites based on polylactides synthesized using plant extracts.

The aim of this work is the green synthesis of silver-containing nanocomposites based on polylactide (PLA) in two ways: reduction of Ag^+^ ions to silver nanoparticles with their subsequent introduction into PLA (mechanical method) and reduction of Ag^+^ ions in polymer matrix PLA-AgPalm (in situ method), as well as the study of the relationship between the structure, morphology, antimicrobial and antiviral properties of nanocomposites created by these two methods.

## 2. Experimental

### 2.1. Materials

The following agents have been used for the production of polymer systems based on polylactide and silver nanoparticles: PLA in the form of a filament (MonoFilament, Ukraine, with the average molar mass *M_w_* = 274,000 g/mol) and water-alcohol extract (70% EtOH) of green tea (brand “Ahmad”). Silver palmitate (AgPalm) was synthesized according to the method described in [30].

### 2.2. Preparation of Polymer Systems

Nanocomposites were formed in the first way by reducing Ag^+^ ions to silver nanoparticles using green tea extract with their subsequent introduction into the polymer matrix of polylactide (mechanical two-stage method). In the first stage, silver nanoparticles were synthesized separately. To 0.2 ± 0.002 g of AgPalm suspended in 25 ± 0.01 mL of chloroform, 4 ± 0.01 mL of 70% ethanol extract of green tea was added and stirred on a magnetic stirrer at T = 60 ± 1 °C for t = 3 h. During the heating process, silver ions were reduced by organic compounds (polyphenols and organic acids) contained in the extract, and the solution became dark brown. The precipitate of silver formed was washed with chloroform and distilled water and dried at T = 70 ± 1 °C. In the second stage, silver nanoparticle powder was added to a solution of 1 ± 0.002 g of PLA in 20 ± 0.01 mL of chloroform (mass ratio of Ag: PLA was 1:99, 2:98, and 4:96). The resulting mixture was stirred on a magnetic stirrer at T = 60 ± 1 °C for t = 30 min, then subjected to ultrasonic treatment at a frequency of 53 kHz and a temperature of T = 45 ± 1 °C for t = 15 min and poured on a glass surface and dried in an oven at T = 50 ± 1 °C for 8 h.

Nanocomposites were synthesized by the second method by reduction of Ag^+^ ions using green tea extract in the PLA-AgPalm polymer matrix (in situ method). To do this, 1 ± 0.002 g of PLA was dissolved in 20 ± 0.01 mL of chloroform, and a predetermined amount of silver palmitate and aqueous-alcoholic solution of green tea extract (based on 2% of dry extract residue in the final composite) was added and stirred at T = 60 ± 1 °C for t = 30 min with a magnetic stirrer and treated by ultrasound at a frequency of 53 kHz at T = 45 ± 1 °C for t = 15 min. Due to the silver ion reduction reaction, the reaction mixture became dark brown. The resulting mixture was poured onto a glass surface and dried in an oven at T = 50 ± 1 °C for 8 h.

As a result, polymer films with a silver content of φ = 1, 2 and 4 wt.% were obtained by these two methods.

### 2.3. Experimental Methods

#### 2.3.1. X-ray Diffraction Pattern

The structure of nanocomposites was investigated by the method of wide-angle X-ray diffraction on the XRD-7000 diffractometer (Shimadzu, Tokyo, Japan), the X-ray optical scheme of which was performed by the Debye-Scherrer method on the transmission of the primary beam through the sample, using CuKα radiation and graphite monochromator at temperatures T = 20 ± 2 °C.

#### 2.3.2. TEM Analysis

The size of the Ag nanoparticles and their distribution in the polymer matrix was examined using a JEM-1230 transmission electron microscope (JEOL, Tokyo, Japan) at the resolution of 0.2 nm. The fractional composition of silver nanoparticles by their size was determined using the ImageJ program.

#### 2.3.3. Thermal Stability by Thermogravimetric Analysis

The thermal decomposition of samples was studied using TGA Q50 (TA Instruments, New Castle, DE, USA) under air atmosphere in the range 20–500 °C at a heating rate of 20 °C/min.

#### 2.3.4. DSC Analysis

Thermal transitions of samples were analyzed by differential scanning calorimetry (DSC) in air atmosphere using a DSC Q2000 apparatus (TA Instruments, New Castle, DE, USA) at the temperature from 20 to 200 °C and a heating rate of 20 °C/min. The degree of crystallinity χ_C_ (%) of the PLA and its nanocomposites was calculated as [31]:

$$\chi_{C}(\%)=\Delta H_{m}/(1-\varphi )\Delta H_{m}^{0}\times 100\%$$

where ΔH_m_ is the melting enthalpy of the samples; $\Delta H_{m}^{0}$ is the melting enthalpy of a 100% crystalline PLA (93 J/g); φ is content of filler in polymer composite (in mass fractions of a unit).

#### 2.3.5. Antimicrobial Activity

The antimicrobial activity of PLA-Ag nanocomposites was tested against reference strains of gram-positive bacterium *Staphylococcus aureus* ATCC 6538, gram-negative bacteria *Escherichia coli* ATCC 35218; and *Pseudomonas aeruginosa* ATCC 27853 and *Candida albicans* ATCC 885–653 as the fungal pathogen [32]. The study was carried out by agar disk diffusion method on nutrient medium LB (Luria-Bertani) (pH 7.0 ± 0.2). Agar plates were inoculated with 10 µL of test microorganisms *S. aureus*, *E. coli*, *P. aeruginosa* (2.5 × 10^5^ CFU/mL), and *C. albicans* (4 × 10^5^ CFU/mL). Then, nanocomposite films (10 mm in diameter) were placed on the agar surface. The plates were incubated at 37 °C for 24 h. The presence of a clear zone free of microorganisms around the films was an indicator of antimicrobial activity. The diameters of inhibition growth zones (mm) were measured. The control was the PLA or PLA-green tea extract film. The experiment was performed in triplicate, and the results are expressed as means ± the standard errors of the means. A Student’s *t*-test was used to compare these results. *p* values lower than 0.05 were considered significant.

#### 2.3.6. Viruses and Cell Culture

In this study, MDCK (Madin-Darby canine kidney) and Hep-2 (human larynx epithelioma cancer) cells were used for cytotoxicity research. For aniviral research, the influenza A virus (IAV) H1N1, strain A/FM/1/47, and human adenovirus serotype 2 (HAdV-2) were used. All epithelial cells were maintained in a sterile plastic falcon (Bioswisstec, Schaffhausen, Switzerland) in a growth medium composed of 45% DMEM (Biowest, Nuaillé, France), 45% RPMI 1640 (Biowest, Nuaillé, France), and 10% fetal bovine serum (FBS, Biowest, Nuaillé, France) heat-inactivated at 56 °C with antibiotics penicillin-streptomycin, 100 μg/mL (Biowest, Nuaillé, France).

#### 2.3.7. The Cytotoxicity Assay

The cytotoxicity of nanocomposites was performed using the MTT test, which is based on determining the functioning of the dehydrogenase activity of mitochondria. The study procedure included 24 h of exposure of the plates in a growth medium for cell cultures at 37 °C. The medium was then added to the monolayer of cell cultures at dilutions of 1 (sample without dilution), 1:10, 1:100, and 1:1000. The analysis of cells was performed at 48 h by the MTT-test described above [33].

#### 2.3.8. The Antiviral Assay

The study nanocomposites were placed in the wells of a 96-well plate (darker side up); 50 μL of undiluted virus suspension was applied on top and incubated at 37 °C for 60 min. Cells were infected with ten-fold serial dilutions of virus-containing material at 50 μL per well. A suspension of the virus was used as a control, and kept in similar conditions without contact with composites. Non-virus infected cells were used as cell controls. The plate was kept in an atmosphere of 5% CO_2_ at 37 °C until the appearance of a pronounced cytopathic effect of the virus. The analysis was performed using the MTT method for HAdV-2 cells and crystal purple staining for the influenza virus. The percentage of cytopathic action of the virus (CPE) and the titer of the virus (TCID_50_/mL) was determined by the formula described in [34]. Antiviral activity of each nanocomposite was determined as the logarithm reduction factor (log_10_TCID_50_/mL) of the viral titer (study samples) compared with untreated infected controls (viral controls). Values were expressed as titer (TCID_50_/mL) and were considered positive if greater or equal to 2 log_10_TCID_50_/mL. The data shown are the mean ± SD from three replicated experiments.

## 3. Results and Discussion

### 3.1. Structural Characterization by X-ray Diffraction

**Figure 1:**
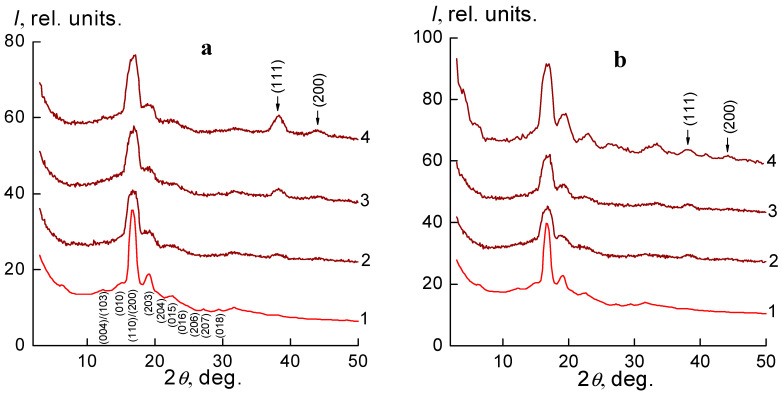
Wide-angle X-ray diffractograms (**a**) PLA (1), and silver-containing nanocomposites obtained by the mechanical method: PLA-1 wt.% Ag (2), PLA-2 wt.% Ag (3), PLA-4 wt.% Ag (4), (**b**) PLA (1), and nanocomposites obtained by in situ method: PLA-1 wt.% Ag (2), PLA-2 wt.% Ag (3), PLA-4 wt.% Ag (4).

Analysis of the wide-angle X-ray diffraction patterns has shown that the pure PLA has a semi-crystalline structure (Figure 1a, curve 1). This is indicated by the corresponding diffraction maxima at 2*θ_m_*~12.4°, 14.9°, 16.8°, 19.2°, 20.8°, 22.4°, 24.1°, 25.0°, 27.4°, and 29.2°, which correspond to the crystallographic planes of Miller indices (004)/(103), (010), (110)/(200), (203), (204), (015), (016), (206), (207) and (018), relating to the crystalline α form PLA [35,36].

During the formation of silver-containing nanocomposites by the introduction of 1, 2 and 4 wt.% silver nanoparticles into the polymer matrix of polylactide (mechanical method) two diffraction maximums appear in the X-ray diffraction patterns at 2*θ_m_*~38.2° and 43.8°, which correspond to crystallographic planes of Miller indices (111) and (200) of the fcc lattice of Ag, respectively (see Figure 1a, curves 2–4). In the synthesis of nanocomposites by the reduction of Ag^+^ ions with green tea extract in the PLA-AgPalm polymer matrix (in situ method), X-ray diffraction patterns also show diffraction maxima which characterize the structure of silver metal but are of much lower intensity, indicating the formation of smaller nanoparticles. As the concentration of nanoparticles in silver-containing nanocomposites obtained by the two methods increases, the intensity of diffraction maxima that characterize the structure of metallic silver increases (Figure 1a,b, curves 2–4).

### 3.2. TEM Analysis

**Figure 2:**
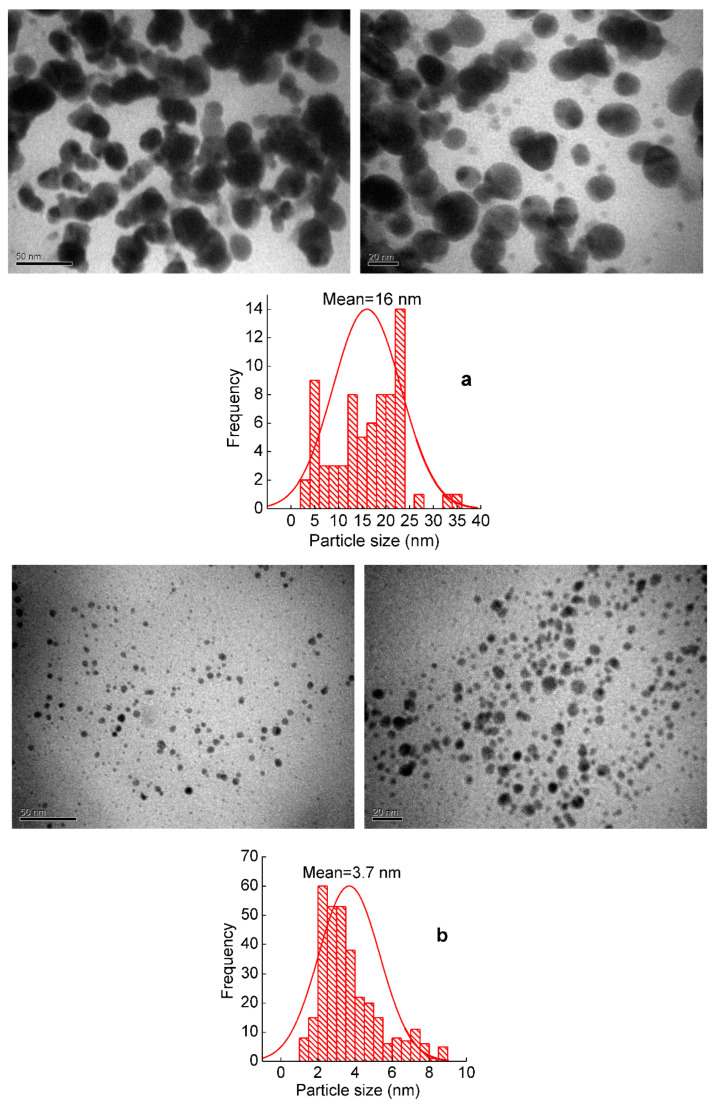
TEM images and histogram of the PLA-4 wt.% Ag nanocomposite obtained by the mechanical method (**a**) and the in situ method (**b**).

From the analysis of the microphotographs it was found that when mechanically filling the polylactide matrix with silver nanoparticles, their average size is 16 nm (Figure 2a). When forming silver-containing nanocomposites by the in situ method, the average size of nanoparticles is much smaller at 3.7 nm, and the size distribution of nanoparticles is narrower (Figure 2b). The figures contain histograms that reflect the fractional composition of silver nanoparticles determined from the analysis of the TEM images. The shape of nanoparticles in nanocomposites obtained in different ways is close to spherical (Figure 2a,b). This effect is due to the fact that in the synthesis of silver nanoparticles separately, followed by their subsequent introduction into the polymer matrix, and then owing to the van der Waals attraction Forces, they are capable of aggregation. When the formation of silver nanoparticles takes place in the polymer matrix of PLA (in situ method), it prevents the aggregation of particles.

### 3.3. Thermal Stability by Thermogravimetric Analysis

**Figure 3:**
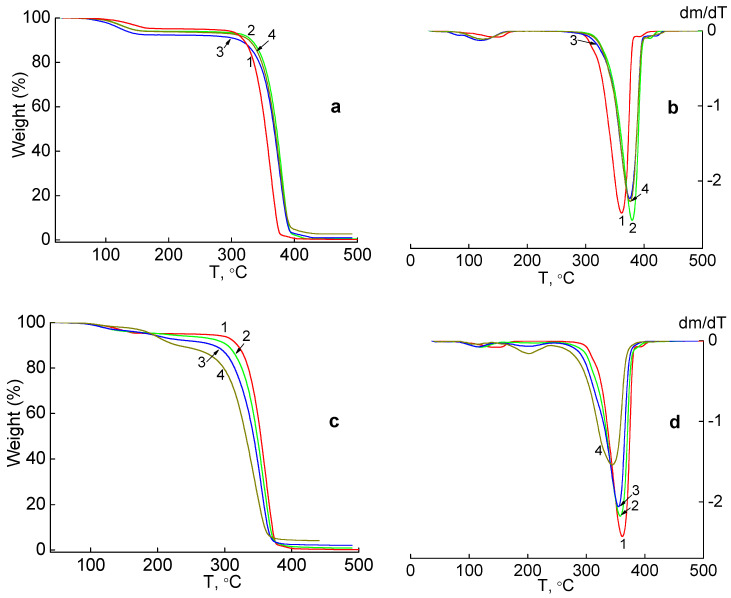
TGA (**a**,**c**) and DTG (**b**,**d**) curves of PLA and silver containing nanocomposites: PLA (1), PLA-1 wt.% Ag (2), PLA-2 wt.% Ag (3), PLA-4 wt.% Ag (4), obtained by the mechanical method (**a**,**b**) and the in situ method (**c**,**d**).

Figure 3a–d show the TGA and differential TGA (DTGA) curves, respectively, for pure PLA and nanocomposites with different contents of silver nanoparticles: 1, 2 and 4 wt.% by weight, obtained by the mechanical method. In the sample of the pure PLA (Figure 3a, curve 1), the weight loss occurs in the first stage in the temperature range of 100–180 °C and is 4.8%. From the DTGA curve it is seen that the maximum corresponding to this process is fixed at T = 145 °C (Figure 3b, curve 1). A characteristic feature of the TGA curves of silver-containing nanocomposites based on PLA is the decrease in mass in the temperature diapason 60–160 °C in the range of 5.8–7.5% for samples with different content of silver nanoparticles (Figure 3a, curves 2–4). On the DTGA curves of these samples there is a peak that shows the maximum rate of this process at T = 121–125 °C (Figure 3b, curves 2–4). It can be assumed that the weight loss of the samples in this temperature range is due to the removal of solvent residues when heated. As can be seen from the TGA/DTGA curves (Figure 3a,b), the thermal properties of PLA-based nanocomposites with a silver nanoparticle content of 1–4 wt.% are similar (Table 1).

In the second stage, the beginning of thermodestruction of the pure PLA film corresponds to the temperature T_onset_ = 323 °C and the main maximum on the DTGA curve, which shows the highest rate of thermodestruction, and it was recorded at T = 362 °C. As can be seen from Table 1, the temperature at the beginning of thermal destruction is the same for all compositions of nanocomposites and is equal to T_onset_ = 340 ± 1.5 °C, which indicates a high thermal stability of the samples. The maximum rate of thermal destruction for the studied silver-containing nanocomposites is also close and is fixed at a temperature of T_max_ = 378 ± 2.5 °C. Table 1 shows that the lowest heat resistance is shown by pure PLA. That is, the introduction of Ag nanoparticles in the PLA matrix increases the heat resistance of PLA-Ag composites.

Figure 3c,d show the TGA and DTGA curves for nanocomposites with different contents of silver nanoparticles: 1, 2 and 4 wt.% (curves 2–4), synthesized by reduction of Ag^+^ ions using green tea extract in a PLA-AgPalm polymer matrix (in situ method). The introduction of 1 wt.% nanosilver into the polylactide matrix shifts the temperature maximum of the first stage, which can be associated with solvent evaporation, from 145 °C to 121 °C (Figure 3c,d, curve 2). Increasing the silver content to 2 wt.% (curve 3) leads to the appearance of the second maximum at T = 202 °C (which can be associated with the second stage of thermal destruction), while the temperature of the first maximum is shifted to 115 °C.

Weight loss in both temperature ranges is 3.2 and 4.7%. The second maximum at T = 202 °C on the curve DTGA is even clearer when the nanosilver content increases to 4 wt.% (curve 4), while the first maximum at T = 116 °C weakens. The weight loss at the second stage corresponds to the destruction of hydrocarbon chains from silver palmitate, the decomposition temperature of which is 244–248 °C [30].

This complex nature of the weight loss of the samples indicates a significant change in the structure of the polymer matrix of the PLA in the presence of smaller silver nanoparticles obtained in situ compared to larger silver nanoparticles that were mechanically introduced into the PLA. Data on mass losses and temperature of the corresponding maxima are shown in Table 2.

In the third stage, when the silver nanoparticles are introduced into the polymer matrix of the PLA, the temperature of the beginning of thermodestruction and the peak temperature on the DTGA curve, which corresponds to the maximum rate of thermodestruction, decrease. Table 2 shows that the highest heat resistance is demonstrated by pure PLA.

**Figure 4:**
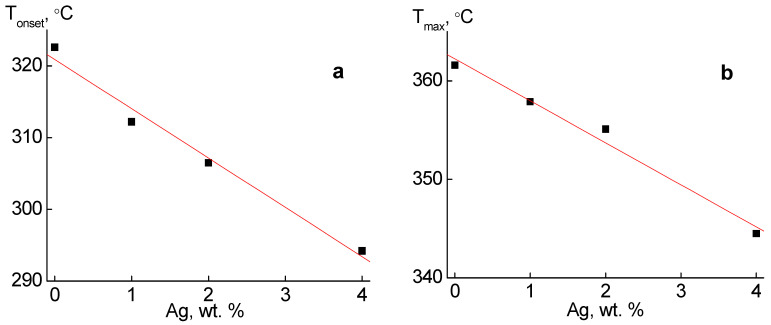
Dependence of temperature of the beginning of thermal destruction T_onset_ (**a**) and the maximum rate of thermal destruction T_max_ (**b**) on the content φ of silver nanoparticles.

The dependence of the temperature of the beginning of destruction (T_onset_) and the peak temperature on the curve DTGA (T_max_) of silver-containing nanocomposites obtained by in situ method on the content of nanoparticles is shown in Figure 4. For both temperatures this dependence is linear and is described by linear equations:

$$T_{\mathrm{onset}}=T_{\mathrm{onset}}^{0}-k_{1}\varphi$$

$$T_{\max}=T_{\max}^{0}-k_{2}\varphi$$

where $T_{\mathrm{onset}}^{0}$ and $T_{\max}^{0}$ are the temperatures of the corresponding parameters for pure PLA, and φ is the concentration of silver nanoparticles (wt.%). The value of the coefficient k in the equations is k_1_ = 7.06 and k_2_ = 4.23. This dependence (Figure 4) indicates a strong interaction of silver nanoparticles with the polymer matrix of the PLA, which leads to a decrease in the heat resistance of the samples.

Thus, in the case of silver containing composites with a mechanical method of introducing Ag nanoparticles into PLA, the addition of silver nanoparticles increases the thermal stability of composites, while in composites obtained by the in situ method, the presence of silver nanofiller reduces the thermal stability of composites. The pattern of TGA/DTGA curves is different for the composites obtained by these two methods.

### 3.4. DSC Analysis

**Figure 5:**
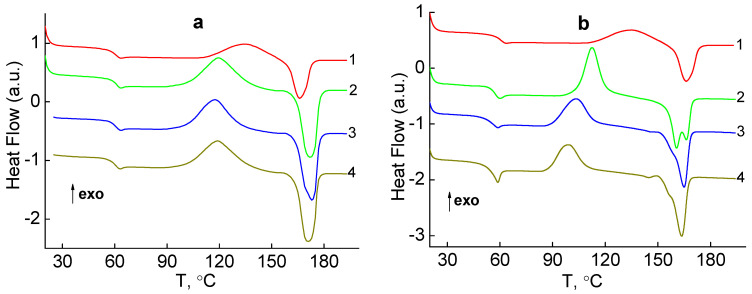
DSC thermograms (**a**) PLA (1), and silver-containing nanocomposites obtained by the mechanical method: PLA-1 wt.% Ag (2), PLA-2 wt.% Ag (3), PLA-4 wt.% Ag (4), (**b**) PLA (1) and nanocomposites obtained by the in situ method: PLA-1 wt.% Ag (2), PLA-2 wt.% Ag (3), PLA-4 wt.% Ag (4).

Figure 5a shows the DSC curves of the samples of pure PLA and nanocomposites with different contents of silver nanoparticles: 1, 2 and 4 wt.%, obtained by the mechanical method. On the DSC curves there are three structural transitions, which are manifested by increasing the temperature of the sample:

1—glass transition in the region of T_g_ = 57–62 °C;
2—cold crystallization in the region of T_ccr_ = 90–150 °C;
3—melting in the region of T_m_ = 160–185 °C.

The parameters of these three transitions for nanocomposites with different contents φ of silver nanoparticles are presented in Table 3.

It should be noted that all three transitions on the studied samples are manifested at the second run, while at the first run only the endothermic peak, which characterizes the melting process, is recorded on the DSC curves. This indicates that pure PLA and nanocomposites based on it with different content of silver nanoparticles have a semi-crystalline structure with a high degree of crystallinity. When the samples are first heated, the effect of the thermal history of the material is leveled, and upon further cooling they acquire structure with a low degree of crystallinity. This is indicated by the character of the curves of the second heating, which shows the glass transition temperature T_g_ of the amorphous phase, and then the temperature of cold crystallization T_ccr_ (Figure 5a, curves 1–4). The presence of a large peak of cold crystallization of the samples during the second heating indicates that a significant part of the amorphous phase in the polymer is ordered and again converted into a crystalline phase. Upon further heating, this crystalline phase melts, which gives a clear endothermic melting peak on the DSC curves (curves 1–4).

The analysis of the data in Table 3 shows that the specific heat of crystallization ΔH_ccr_ and the specific heat of melting ΔH_m_ of pure PLA and silver-containing nanocomposites are close. This indicates that at the beginning of the second run, the studied samples had a low degree of crystallinity, i.e., their structure was almost amorphous, and the crystalline phase, which melted, was formed during cold crystallization. The degree of crystallinity χ_C_, calculated taking into account the value of melting heat = 93 J/g for fully crystalline PLA, is approximately the same for all nanocomposites and is in the range of χ_C_ = 37.6–36.0%. The authors of [37] consider the degree of crystallinity acquired as a result of the heating as the maximal, compared to the minimal that exists before heating. Table 3 shows that all parameters of nanocomposites based on pure PLA with different content of silver nanoparticles are approximately the same, i.e., the introduction of nanoparticles into the polymer matrix in the range of φ = 1–4 wt.% does not significantly affect the characteristics of the amorphous phase and crystalline phase of the PLA. Thus, the DSC results show that the variation of content of the silver nanoparticles is not manifested in the structure formation of silver-containing nanocomposites obtained in the mechanical manner.

Figure 5b shows the DSC curves of nanocomposites with different contents of silver nanoparticles: 1, 2 and 4 wt.%, synthesized by reduction of Ag^+^ ions using green tea extract in the polymer matrix PLA-AgPalm in situ. The DSC curves of these samples also show three structural transitions, namely:

1—glass transition in the region of T_g_ = 54–61 °C;
2—cold crystallization in the region of T_ccr_ = 99–136 °C;
3—melting in the region of T_m_ = 158–167 °C.

The parameters of the processes of glass transition, cold crystallization and melting of these nanocomposites are presented in Table 3. The thermal behavior of the studied samples obtained by the in situ method has some differences from the behavior of samples obtained by the mechanical method (Figure 5a,b). As can be seen from Figure 5b when introducing silver nanoparticles into PLA by the in situ method, an endothermic peak appears on the step curve of the glass transition process, the intensity of which increases with increasing nanofiller content and which can be associated with enthalpy relaxation [38,39]. This can be explained by the fact that with increasing content of silver nanoparticles, the contribution of the boundary layer of PLA, which is formed due to the interaction with the surface of the nanofiller, increases. The proportion of the boundary layer in the case of composites obtained in situ is quite large due to the small size of silver nanoparticles (3.7 nm versus 16 nm in composites formed by mechanical means). This layer with an altered structure (usually in the boundary layer, the molecular packaging is less dense than in the volume of the polymer [40]) may be responsible for the effects of enthalpy relaxation.

Figure 5b shows that in the studied samples during the second run above the glass transition temperature, cold crystallization of the PLA begins. As in the composites obtained by the mechanical method, the specific heat of crystallization ΔH_ccr_ and the specific heat of melting ΔH_m_ of silver-containing nanocomposites are close (Table 3). Thus, the molten crystalline phase was formed during cold crystallization.

Analysis of DSC thermograms in the melting region shows that with the introduction of Ag nanoparticles in the PLA, the degree of crystallinity χ_C_ increases and is in the range of 32–27.7%, compared with 19.5% in pure PLA (Table 3), which indicates the nucleating action of silver nanoparticles. The appearance of a low-temperature shoulder at the melting peak on the thermograms of samples containing 1, 2 and 4 wt.% silver nanoparticles (Figure 5b, curves 2–4, Table 3) is worthy of note. This effect can be attributed to the presence of a boundary layer with a distended structure, which affects the formation of the crystalline phase of the PLA.

In silver-containing nanocomposites obtained by mechanical filling of silver nanoparticles of average size d = 16 nm, the filler content does not affect the glass transition temperature and melting parameters. In nanocomposites formed by the in situ method, in which the average size of silver nanoparticles is d = 3.7 nm, the glass transition temperature decreases with increasing silver content, and the appearance of a low-temperature shoulder at the melting peak is observed. Obviously, this difference is related to the size of the nanoparticles and the value of their specific surface area, which determines the amount of polymer included in the boundary layer. The specific surface area S_sa_ of nanoparticles was evaluated by the ratio [41]:

$$S_{\mathrm{sa}}=\frac{6}{\rho \cdot d}$$

where ρ is the density of silver, and d is the size of the nanoparticles. The calculation according to this equation showed that S_sa_ = 36 m^2^/g for nanoparticles that were mechanically introduced into the PLA (d = 16 nm), and S_sa_ = 155 m^2^/g for nanoparticles obtained in situ (d = 3.7 nm). These data indicate significant differences between the studied series of nanocomposites in the degree of interaction of the nanofiller with the PLA matrix and in the amount of polymer in the boundary layer of the PLA and, accordingly, in the behavior of composites during glass transition and melting.

**Figure 6:**
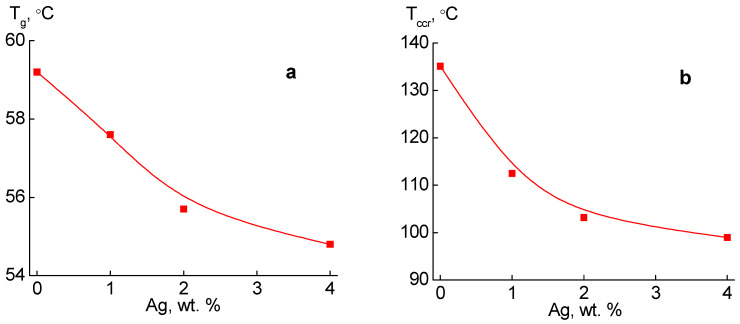
Dependence of glass transition temperature (**a**) and crystallization (**b**) on the content of silver nanoparticles.

Figure 6a shows the dependence of the glass transition temperature of the samples on the content of silver nanoparticles in the PLA. The dependence is nonlinear, and the greatest effect on the glass transition temperature of the PLA is the addition to the PLA 1 and 2 wt.% Ag; with a further increase in the content of the filler, its effect decreases. Thus, the boundary layer on the surface of the filler with distended molecular packaging provides greater mobility of the segments of macromolecules, which reduces the glass transition temperature of the samples. It should be noted that the effect of the nanofiller on the value of T_g_ can be different depending on the role it plays, for example in [42,43] a linear increase in the glass transition temperature with increasing filler content was observed, where its interaction with the polymer matrix led to the creation of the complexes and restrictions in molecular mobility.

Also, the cold crystallization temperature Tccr of the studied samples shifts to the low-temperature region with increasing nanofiller content (Figure 6b). Obviously, greater molecular mobility in silver-filled nanocomposites promotes the process of cold crystallization at lower temperatures.

### 3.5. Antimicrobial Activity of PLA-Ag Nanocomposites

In the study of silver-containing nanocomposites, which were obtained by the mechanical method: PLA-1 wt.% Ag, PLA-2 wt.% Ag, and PLA-4 wt.% Ag antimicrobial activity against the studied test cultures of microorganisms *S. aureus*, *E. coli*, *P. aeruginosa* and *C. albicans* were not detected. After 24 h of incubation, the zones of inhibition of test cultures around the disks were not observed, and intensive growth of test cultures was recorded. This result may be due to the larger size of the nanoparticles, which have less chemical activity compared to nanoparticles obtained in situ.

**Figure 7:**
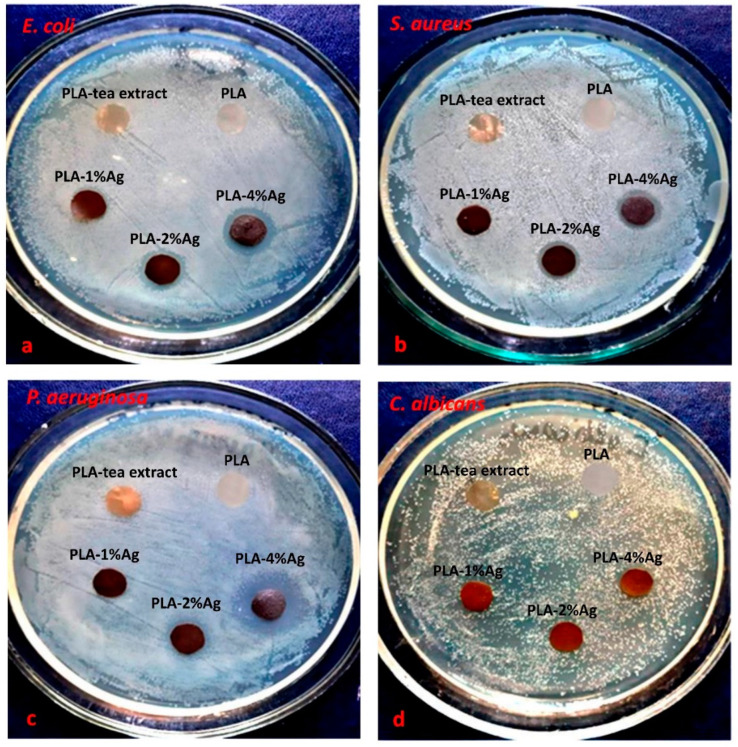
Images of antimicrobial tests of PLA-Ag nanocomposites obtained by the in situ method against the studied test cultures of microorganisms (**a**) *E. coli*, (**b**) *S. aureus*, (**c**) *P. aeruginosa* and (**d**) *C. albicans*. The concentration of nanoparticles in wt.%.

The nanocomposites synthesized by the reduction of Ag^+^ ions with green tea extract in the polymer matrix PLA-AgPalm (in situ method) with silver concentrations φ = 1, 2, and 4 wt.% showed antimicrobial activity against the test cultures of *S. aureus*, and *E. coli*. Thus, after 24 h of incubation on the plates with test cultures, the presence of a clear zone of inhibition of microorganisms was observed (Figure 7a,b). The studied nanocomposites showed the highest activity at the concentration of nanosilver φ = 4 wt.%. The diameters of the zones of inhibition growth were 11.9 ± 0.8 mm relative to *S. aureus* and 13.1 ± 1.4 mm relative to *E. coli*, while at the concentration of silver nanoparticles φ = 1 and 2 wt.% the activity was lower (Table 4). The antimicrobial activity of silver-containing nanocomposites against *P. aeruginosa* was detected in samples with nanosilver concentrations φ = 2, and 4 wt.% (Figure 7c), and the inhibition zones were 10.9 ± 0.6 mm and 14.9 ± 1.2 mm, respectively. The studied nanocomposites PLA-Ag had the lowest antimicrobial activity against the fungal pathogen of *C. albicans*. The diameter of the zone of inhibition growth for samples with silver concentration φ = 4 wt.% were 10.6 ± 0.7 mm (Figure 7d). In the control films, which did not contain silver nanoparticles, active growth of test microorganisms and the absence of a zone of inhibition were observed (Figure 7, Table 4).

The antimicrobial activity of silver nanoparticles has been reported in many research papers. However, the mechanism of antibacterial action of these compounds has not been elucidated yet. Silver nanoparticles were proven to exert the same effect against both gram-positive/negative bacteria and fungi, and need to reach the cell membrane to achieve an antimicrobial effect. It was suggested that silver nanoparticles attach to the surface of the cell membrane and disturb its function, penetrate microorganisms, and release silver ions [44]. Silver nanoparticles’ general mechanism of antimicrobial action is based on the inhibition of cell wall synthesis, protein synthesis mediated by the 30s ribosomal subunit, and nucleic acid synthesis. This also leads to the formation of reactive oxygen species [45].

### 3.6. Antiviral Activity of Ag-Containing Nanocomposites

**Figure 8:**
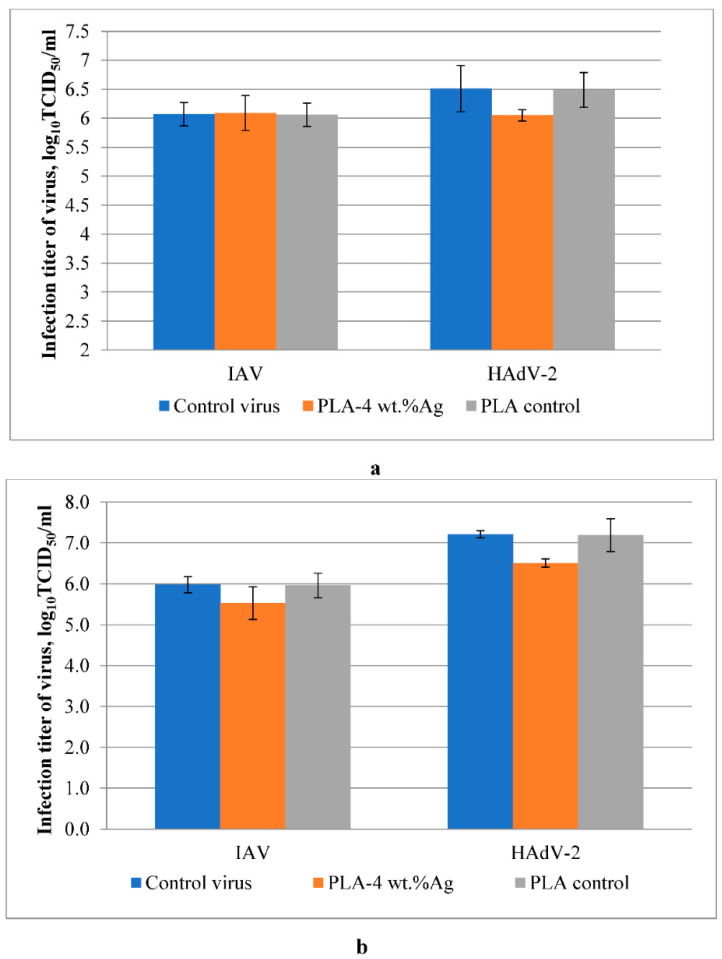
Antiviral activity of PLA-4 wt.%Ag nanocomposites obtained by mechanical method (**a**) and in situ method (**b**) against influenza virus and adenovirus.

As shown in Figure 8a, the silver-containing nanocomposites synthesized by the mechanical method showed a relative virucidal effect against human adenovirus. Inhibiting infectious virus titer by 0.46 log_10_TCID_50_/mL compared to virus control has been established. For the influenza virus, the value of the infectious titer was at the control level.

Figure 8b shows that nanocomposites synthesized by reducing Ag^+^ ions in PLA with green tea extract (in situ method) showed weak virucidal activity in models of influenza virus and human adenovirus serotype 2. A decrease in titer of 0.7 log_10_TCID_50_/mL was detected in the adenovirus model; for the influenza virus, there was an inhibition of the infectious titer of the virus by 0.45 log_10_TCID_50_/mL compared with controls. The PLA control does not show the antiviral effect on influenza and adenovirus. Silver nanoparticles are the most commonly synthesized and investigated materials due to their excellent catalytic, optical, and biological properties. According to the literature, Ag nanoparticles might interact with the pathogenic viruses in extracellular and intracellular ways [46]. The first type of interaction is based on the direct cooperation of Ag nanoparticles with virus particles. In contrast, the second type of interaction included interaction between Ag nanoparticles and various viral and cellular factors responsible for reproduction and the release of progeny virions [47,48,49,50].

Ag nanoparticles in the studied systems based on PLA were synthesized by the mechanical and in situ methods. As described above, mechanically prepared nanocomposites have larger nanoparticles than nanocomposites obtained by the in situ method. According to the results of virucidal studies, smaller nanoparticles were active against adenovirus and influenza, whereas larger nanoparticles had a virucidal effect only against the adenovirus. Analyzed data showed that Ag nanoparticles on the surface lead to loss of infectivity of enveloped and non-enveloped viruses.

### 3.7. Cytotoxicity of Ag-Containing Nanocomposites

**Figure 9:**
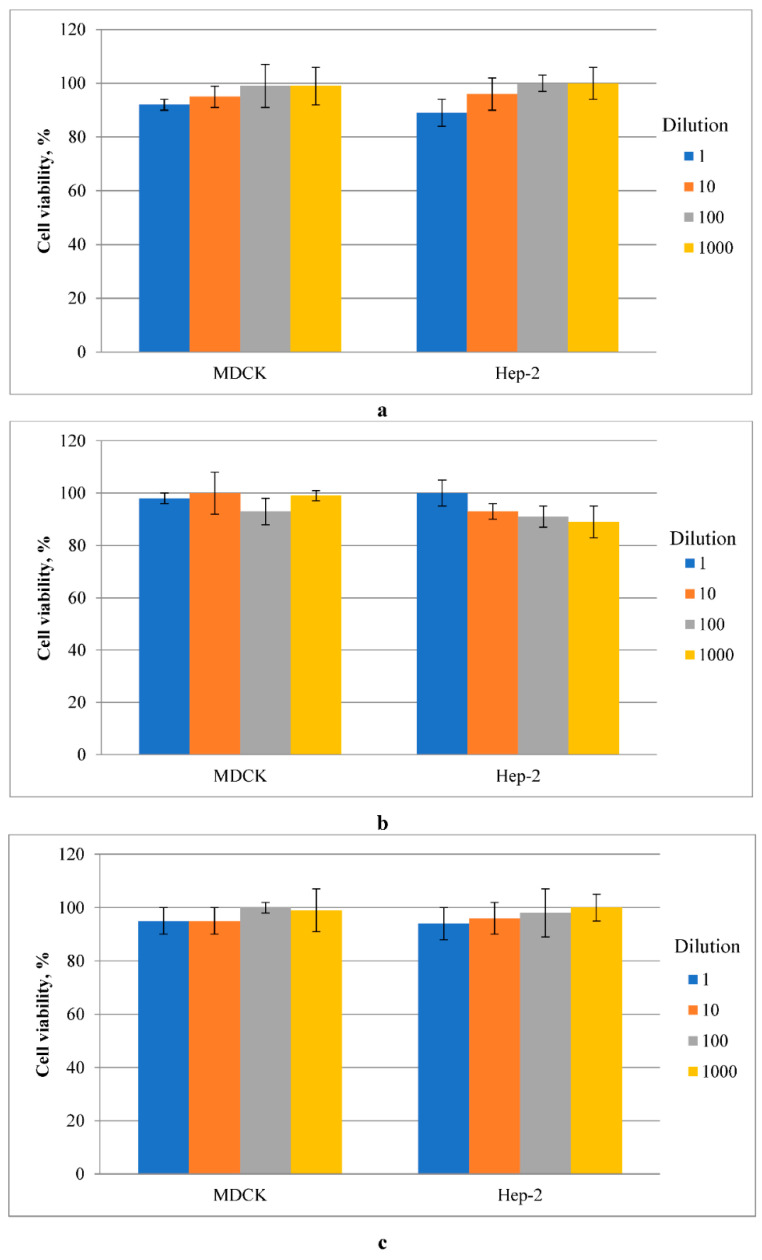
Cytotoxicity of PLA-4 wt.%Ag nanocomposites obtained mechanical method (**a**) and in situ method (**b**) and PLA control sample (**c**) in MDCK and Hep-2 cells.

It was found that silver-containing nanocomposites based on polylactide obtained by the mechanical method have a weak inhibitory effect in cell cultures of MDCK and Hep-2. As shown in Figure 9a, the cytotoxic effect has a dose-dependent impact, so a decrease in the inhibitory effect on both epithelial cultures was detected with more significant dilution. However, it should be noted that the percentage of living cells ranged from 89 to 100% in the range of the studied dilutions, so it can be argued that these samples are not toxic.

As shown in Figure 9b, nanocomposites obtained in situ did not have a cytotoxic effect on any studied cell cultures. Thus, the MDCK cell viability ranged from 93 to 100%. In Hep-2 cells under conditions of incubation with PLA-4 wt.% Ag films, with increasing dilution, the percentage of living cells decreased from 100 to 88%. Still, these results show that nanocomposites obtained by the in situ method do not have a cytotoxic influence on the epithelial cultures. The PLA control (the basic of nanocomposites) was non-toxic at both epithelial cell cultures, and cell viability was nearly 100%.

## 4. Conclusions

In this study, two methods of the green synthesis of nanocomposites based on polylactide (PLA) and Ag nanoparticles were developed. The first method used green tea extract to reduce Ag^+^ ions from silver palmitate (AgPalm) to silver nanoparticles and then introduced them into the PLA (mechanical method). According to the second method, Ag^+^ ions were reduced in the polymer matrix of PLA-AgPalm (in situ method). The relationship between the structure, morphology, antimicrobial and antiviral properties of silver-containing nanocomposites synthesized in different ways has been established. The reduction of Ag^+^ ions to metal nanoparticles of bigger size d = 16 nm (mechanical method) and smaller size d = 3.7 nm (in situ method) was confirmed by the WAXS and TEM methods.

Data of thermogravimetric analysis showed a strong effect of smaller silver nanoparticles on the structure of the PLA matrix; with increasing content φ of silver nanoparticles in the polymer matrix of PLA the heat resistance of nanocomposites deteriorates. According to the DSC data in the nanocomposites with a smaller size of nanoparticles with increasing silver content the glass transition temperature decreases. The effect of nanoparticle content on the glass transition temperature was not observed in the samples formed by mechanical means. This is due to the size of nanoparticles and the value of their specific surface area S_sa_, which determines the degree of interaction of the nanofiller with the polymer matrix of PLA. The specific surface area is much higher for nanoparticles obtained in situ (d = 3.7 nm), S_sa_ = 155 m^2^/g, compared with nanoparticles obtained by the mechanical method (d = 16 nm), S_sa_ = 36 m^2^/g.

In the study of silver-containing nanocomposites synthesized by the mechanical method in the polymer matrix of PLA with the concentrations of φ = 1, 2 and 4 wt.%, antimicrobial activity against microorganisms *S. aureus*, *E. coli*, *P. aeruginosa* and *C. albicans* was not detected. Silver-containing nanocomposites formed in situ by the reduction of Ag^+^ ions with green tea extract in the polymer matrix PLA-AgPalm have been shown to exhibit antimicrobial activity against gram-positive bacterium *S. aureus*, gram-negative bacteria *E. coli*, *P. aeruginosa*, and the fungal pathogen of *C. albicans*, with sample activity increasing with increasing nanoparticle concentration. These nanocomposites showed the highest activity at the concentration of nanosilver φ = 4 wt.%. The diameter of the inhibition zone was 11.9 ± 0.8 mm for *S. aureus*, 13.1 ± 1.4 mm for *E. coli*, 14.9 ± 1.2 mm for *P. aeruginosa*, and 10.6 ± 0.7 mm for *C. albicans*. The relative antiviral activity of nanocomposites obtained in two methods against influenza A virus and adenovirus serotype 2 was also revealed. The obtained nanocomposites were not cytotoxic and they did not inhibit the viability of MDCK and Hep-2 cell cultures.

The elaborated materials can be promising for the treatment of wounds of various infectious origins; for the formation of antimicrobial coatings in medical, pharmacological, and biological laboratories to ensure sterile conditions; and for creating packaging materials for the long-term, high-quality and safe storage of food, etc.

## Figures and Tables

**Table 1:** Thermal properties of PLA-Ag nanocomposites obtained by the mechanical method.

Samples	First Stage		Second Stage	
	Weight Loss, %	T_max_, °C (DTG)	T_onset_, °C	T_max_, °C (DTG)
PLA	4.8	145	323	362
PLA-1 wt.% Ag	5.8	125	341	380
PLA-2 wt.% Ag	7.5	121	339	375
PLA-4 wt.% Ag	6.0	124	340	376

**Table 2:** Thermal properties of PLA and PLA-Ag nanocomposites obtained by the in-situ method.

Samples	First Stage		Second Stage		Third Stage	
	Weight Loss, %	T_max_, °C (DTG)	Weight Loss, %	T_max_, °C (DTG)	T_onset_, °C	T_max_, °C (DTG)
PLA	4.8	145	–	–	323	362
PLA-1 wt.% Ag	4.5	121	–	–	312	358
PLA-2 wt.% Ag	3.2	115	4.7	202	306	355
PLA-4 wt.% Ag	1.8	116	8.8	202	294	344

**Table 3:** DSC data of PLA and PLA-Ag nanocomposites obtained by mechanical and in situ method.

Specimen	Glass Transition		Cold Crystallization		Melting		χ_C_, %
	T_g_, °C	ΔC_p_, J/g·°C	T_ccr_, °C	ΔH_ccr_, J/g	T_m_, °C	ΔH_m_, J/g	
PLA	59.2	0.412	135.1	16.74	166.1	18.12	19.5
PLA-1 wt.% Ag (mech. method)	61.3	0.378	119.7	37.35	172.3	34.21	37.1
PLA-2 wt.% Ag (mech. method)	61.2	0.432	117.6	34.71	173.2	32.83	36.0
PLA-4 wt.% Ag (mech. method)	60.5	0.443	119.0	35.0	170.9	33.58	37.6
PLA-1 wt.% Ag (in-situ)	57.6	0.459	112.5	28.5	160/166.4	28.12	32.0
PLA-2 wt.% Ag (in-situ)	55.7	0.449	103.2	22.97	158.4/165.0	23.51	27.7
PLA-4 wt.% Ag (in-situ)	54.8	0.394	99.0	20.35	156.5/163.6	25.09	31.9

**Table 4:** Antimicrobial activity of PLA-Ag nanocomposites obtained by the in situ method.

Polymer Systems	Diameter of Inhibition Zone, mm			
	*S. aureus*	*E. coli*	*P. aeruginosa*	*C. albicans*
PLA	0	0	0	0
PLA-tea extract	0	0	0	0
PLA-1 wt.% Ag	10.7 ± 0.4	11.0 ± 0.5	0	0
PLA-2 wt.% Ag	11.0 ± 0.6	11.8 ± 1.1	10.9 ± 0.6	0
PLA-4 wt.% Ag	11.9 ± 0.8	13.1 ± 1.4	14.9 ± 1.2	10.6 ± 0.7

## Data Availability

All the data of the present study are present in the manuscript.
